# Development of a Unique Small Molecule Modulator of CXCR4

**DOI:** 10.1371/journal.pone.0034038

**Published:** 2012-04-02

**Authors:** Zhongxing Liang, Weiqiang Zhan, Aizhi Zhu, Younghyoun Yoon, Songbai Lin, Maiko Sasaki, Jan-Michael A. Klapproth, Hua Yang, Hans E. Grossniklaus, Jianguo Xu, Mauricio Rojas, Ronald J. Voll, Mark M. Goodman, Richard F. Arrendale, Jin Liu, C. Chris Yun, James P. Snyder, Dennis C. Liotta, Hyunsuk Shim

**Affiliations:** 1 Department of Radiology and Imaging Sciences, Emory University, Atlanta, Georgia, United States of America; 2 Department of Chemistry, Emory University, Atlanta, Georgia, United States of America; 3 Department of Medicine, Emory University, Atlanta, Georgia, United States of America; 4 Department of Ophthalmology, Emory University, Atlanta, Georgia, United States of America; 5 Winship Cancer Institute, Emory University, Atlanta, Georgia, United States of America; 6 The Emory Institute for Drug Discovery, Emory University, Atlanta, Georgia, United States of America; National Taiwan University Hospital, Taiwan

## Abstract

**Background:**

Metastasis, the spread and growth of tumor cells to distant organ sites, represents the most devastating attribute and plays a major role in the morbidity and mortality of cancer. Inflammation is crucial for malignant tumor transformation and survival. Thus, blocking inflammation is expected to serve as an effective cancer treatment. Among anti-inflammation therapies, chemokine modulation is now beginning to emerge from the pipeline. CXC chemokine receptor-4 (CXCR4) and its ligand stromal cell-derived factor-1 (CXCL12) interaction and the resulting cell signaling cascade have emerged as highly relevant targets since they play pleiotropic roles in metastatic progression. The unique function of CXCR4 is to promote the homing of tumor cells to their microenvironment at the distant organ sites.

**Methodology/Principal Findings:**

We describe the actions of N,N′-(1,4-phenylenebis(methylene))dipyrimidin-2-amine (designated MSX-122), a novel small molecule and partial CXCR4 antagonist with properties quite unlike that of any other reported CXCR4 antagonists, which was prepared in a single chemical step using a reductive amination reaction. Its specificity toward CXCR4 was tested in a binding affinity assay and a ligand competition assay using ^18^F-labeled MSX-122. The potency of the compound was determined in two functional assays, Matrigel invasion assay and cAMP modulation. The therapeutic potential of MSX-122 was evaluated in three different murine models for inflammation including an experimental colitis, carrageenan induced paw edema, and bleomycin induced lung fibrosis and three different animal models for metastasis including breast cancer micrometastasis in lung, head and neck cancer metastasis in lung, and uveal melanoma micrometastasis in liver in which CXCR4 was reported to play crucial roles.

**Conclusions/Significance:**

We developed a novel small molecule, MSX-122, that is a partial CXCR4 antagonist without mobilizing stem cells, which can be safer for long-term blockade of metastasis than other reported CXCR4 antagonists.

## Introduction

Chemokines are small, pro-inflammatory cytokines of approximately 10 kDa that orchestrate a diverse set of activities through interaction with their cognate receptors. Coupling of stromal cell derived factor 1 (SDF-1; CXCL12) with its receptor CXCR4, which was previously identified as a major coreceptor for the entry of T-tropic HIV [1], [2], [3], [4], plays critical roles in inflammation [5], as well as cancer metastasis [6]. The invasion of cancer cells into surrounding tissue is associated with considerable destruction and regeneration of intercellular elements [7]. Newly synthesized stroma, known as “cancer-induced stroma,” is composed of inflammatory cells (including lymphocytes, granulocytes, and macrophages), endothelial cells of blood and lymph vessels, pericytes, and fibroblasts. Solid tumors are often infiltrated with leukocytes and macrophages. In some tumors, leukocytes can account for up to 50% of the tumor mass, the most represented subsets being lymphocytes and tumor-associated macrophages (TAMs). The presence of TAM at the tumor site represents one of the hallmarks of cancer associated inflammation [8]. TAMs derive from circulating monocytes that are selectively attracted within the tumor microenvironment by locally produced chemotactic factors, such as CXCL12. Incoming monocytes differentiate in the tumor microenvironment to tissue resident macrophages. In turn, several TAM products have been shown to directly stimulate the growth of tumor cells. Additionally, TAMs also contribute to the angiogenic switch by releasing angiogenic factors (VEGF, FGF, and CXCL12) and to the degradation and remodeling of the matrix with metalloproteases (MMPs), suggesting an important role in neovascular formation and subsequent tumor progression [9]. In addition, the CXCR4/CXCL12-axis has been shown to play a pivotal role in trafficking and homing of normal stem cells and metastasis of cancer stem cells to organs that express high levels of CXCL12, such as the lymph nodes, lungs, liver, and bone [10], [11]. Homing, the mechanism that allows foreign tissue-origin cells to reside and proliferate, is believed to be the rate-limiting step of the multi-step metastatic process [12]. Kang et al. generated an animal model of bone metastasis by the intercardiac injection of MDA-MB-231 breast cancer cells into female SCID mice [13]. A subsequent microarray analysis on a sub-population of MDA-MB-231 cells with elevated metastatic activity isolated from the mouse showed that one of the six genes responsible for the metastatic phenotype was CXCR4, which was responsible for homing of breast tissue-origin tumor cells to bone. In samples collected from various breast cancer patients, Muller *et al* [14] found that the level of expression of CXCR4 is higher in primary tumors relative to normal mammary glands or mammary epithelial cells. By contrast, CXCL12 is highly expressed in the most common destinations of breast cancer metastasis including the lymph nodes, lung, liver, and bone marrow. Taken together, CXCR4 and CXCL12 may indeed play critical roles in breast cancer metastasis. Thus, it is important to develop anti-CXCR4 compounds that can intervene in this interaction. The expression of CXCR4 has been detected in 23 different types of cancers, making it the most common chemokine receptor expressed on cancer cells [15]. There is a significant volume of literature demonstrating that CXCR4 and CXCL12 play critical roles in cancer metastasis in numerous types of cancers [16] and it is important to develop anti-CXCR4 compounds to intervene in this progression.

The current clinical lead compound, AMD3100, is a metal-chelating bicyclam that has been shown to block calcium flux induced by CXCR4 activation. Its chelating properties are likely related to the cardiotoxicity reported in its clinical trial against HIV [17]. AMD3100 was recently approved by the FDA for stem cell mobilization. While AMD3100 can benefit patients with certain diseases, it can also induce lung or liver fibrosis [18], and the mobilized cells may act as potential cancer stem cells [19]. Therefore, while limited single use of AMD3100 is widely accepted, drugs with improved toxicity profiles will be required for long term clinical use. We had previously reported that blocking the CXCR4/CXCL12 interaction with a peptidic CXCR4 antagonist, TN14003, suppresses lung metastasis in models of breast cancer [20] and head and neck cancer [21]. In addition, we reported that TN14003 blocks bleomycin-induced lung fibrosis [22]. Although the TN14003 results provided an important proof of concept, its lack of oral availability promted us to look for alternatives that possessed better phamacologic profiles. Using TN14003 as our benchmark, we herein report the development of a novel, small molecule CXCR4 modulator, MSX-122, that specifically blocks selected functions of CXCR4.

Since a large patient population can benefit from anti-metastasis treatment, our drug could potentially have a significant impact on human health.

## Results

### Chemistry

**Figure 1:**
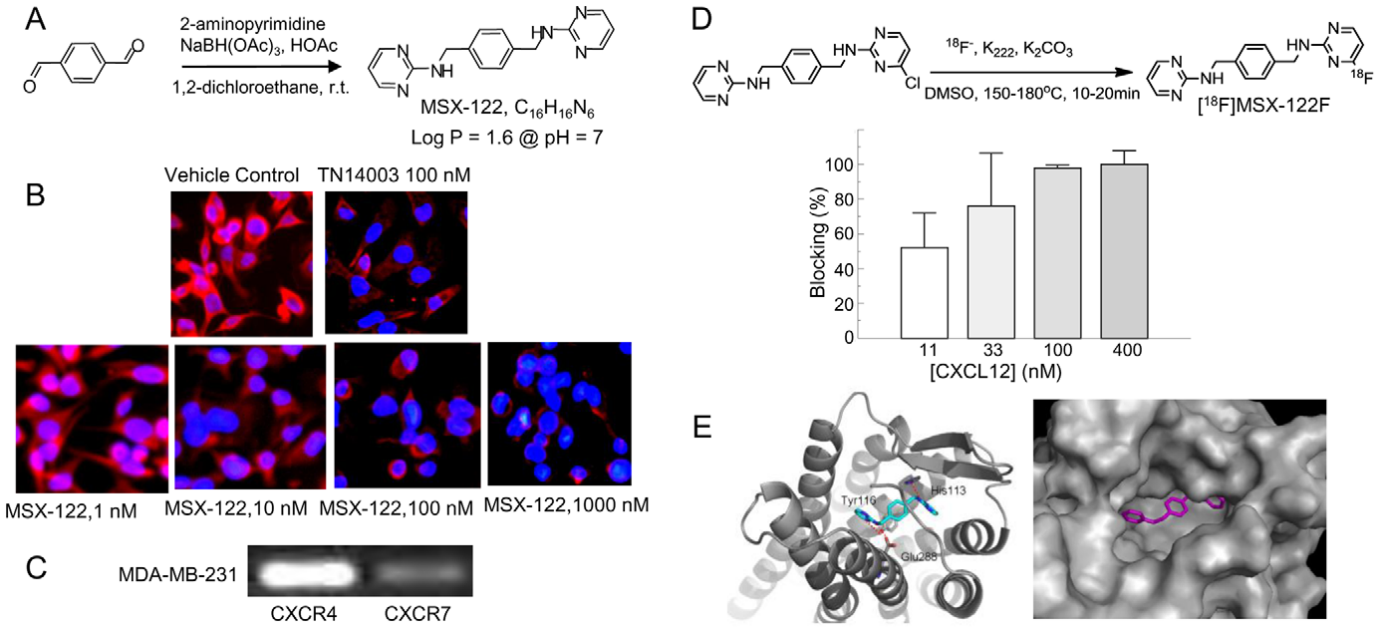
Synthesis of MSX-122 and *in vitro* functional assays. (A) Scheme of MSX-122 preparation. (B) Representative immunofluorescence images show competition-binding assay using the biotinylated CXCR4 antagonist TN14003. MDA-MB-231 cells on an 8-well slide chamber were treated with TN14003 or MSX-122 at various concentrations for 15 minutes at room temperature. The cells were subsequently fixed and incubated with biotin-labeled TN14003 (0.05 µg/ml). After washing, cells were incubated with streptavidin-rhodamine. Red color represents CXCR4. Nuclei were counterstained with cytox blue. (C) RT-PCR results of CXCR4 and CXCR7 reveal that MDA-MB-231 cells do not express CXCR7. (D) Scheme of fluorine-18 labeling of MSX-122 is shown. To determine binding of MSX-122 to a CXCL12 binding site on CXCR4, we tested whether preincubation with CXCL12 (11, 33, 100, and 400 nM) before adding [^18^F]MSX-122F blocked binding to CXCR4 in the CXCR4-positive metastatic SCCHN cells. (E) A low energy predicted binding pose for MSX-122 (magenta) in the CXCR4 X-ray structure (grey) is illustrated by two different visualizations: ribbon (left) and protein surface (right) representations. The complex was constructed by extracting the small molecule antagonist IT1t from the X-ray structure (pdb code 3ODU; [33]) followed by docking MSX-122 into the corresponding binding site.

Initially, we proposed that inclusion of a nitrogen atom in each of the terminal aromatic rings (i.e., pyridyl instead of phenyl) might impede rapid oxidative metabolism and improve inhibitor pharmacokinetic profiles. This working hypothesis was validated in our previously reported structure-activity relationship (SAR) study that led to discovery of WZ811 [23]. A second nitrogen atom substitution in the terminal aromatic rings (i.e., pyrimidyl), which was designated as MSX-122, further improved the pharmacokinetic profile. This compound was prepared in a single chemical step using a reductive amination reaction (**Figure 1A**) [24].

### Screening for Anti-CXCR4 Small Molecules via Selected *in vitro* Assays

For primary compound screening, the previously reported binding affinity assay was utilized [23] (**Figure 1B**). Although CXCL12 can also interact with the CXCR7 chemokine receptor [25], [26], our screening was specific for CXCR4, since the MDA-MB-231 cells used for primary screening express insignificant levels of CXCR7 (**Figure 1C**). Effective concentration for MSX-122 was at a low nanomolar (the concentration at which each compound blocks more than 50% of TN14003 binding on CXCR4).

MSX-122 cannot block ^125^I-labeled full length CXCL12 binding to CXCR4 (data not shown), while the reports about AMD3100 are controversial [27], [28], [29], [30]. This may be due to the fact that CXCL12, a 10 kDa peptide, has multiple interaction sites on CXCR4 [27], [31]. However, we anticipated that CXCL12 would block MSX-122 binding to CXCR4 because MSX-122 binds to a CXCL12 binding site on CXCR4. To determine whether MSX-122 directly binds to the CXCL12 site on CXCR4, we utilized highly sensitive radiotracer technique, using a fluorine-18 radiolabeled MSX-122 analog (MSX-122F) in which one pyrimidine ring of MSX-122 was labeled by fluorine-18 (**Figure 1D**). Then, we tested whether preincubation with CXCL12 before adding [^18^F]MSX-122F blocked binding to CXCR4 in the CXCR4-positive metastatic squamous cell carcinoma of head and neck (SCCHN) cells that we established from a poorly metastatic parental cell line by four rounds of *in vivo* selection using a lymph node metastatic xenograft mouse model. These metastatic SCCHN cell lines expressed high levels of CXCR4 while non-metastatic parental cells did not [21], [32]. As anticipated, inhibition occurred in a dose-dependent manner (**Figure 1D**). Although the MSX-122F is a fluorinated compound, it behaves the same as MSX-122 in all the *in vitro* screening assays employed.

### CXCR4/MSX-122 Docking Model

The published crystallographic structure of CXCR4, bound by the small molecule antagonist IT1t (2.5 Å resolution; PDB code: 3ODU) [33] (Data S2), has been utilized to explore the interaction of MSX-122 with CXCR4 and in comparison to IT1t, a CXCR4 antagonist. Thus, IT1t was removed from the complex, and the flexible ligand Glide docking methodology with extra precision [34], [35], [36] was employed to predict binding poses for MSX-122 in the binding site of 3ODU/CXCR4. The resulting binding poses were subsequently sorted by energy with the MM-GBSA scoring algorithm, which provides an estimate of relative binding free energies [37], [38]. A predicted binding pose of low energy is shown in **Figure 1E**. MSX-122 (magenta) resides in close proximity to the binding locale occupied by a TN14003 analog in the rather spacious pocket. The docking result is consistent with Gupta’s speculation that small molecule antagonists can inhibit functional signal transduction without displacement of CXCL12 because CXCL12 interacts via multiple sites, one being the N-terminal segment of CXCR4 at the receptor surface [27], [31]. Thus, it is not unreasonable to postulate that a large molecule such as TN14003 can displace CXCL12 binding to CXCR4, while MSX-122 near the bottom of the binding pocket cannot. Nonetheless, we postulate that MSX-122 bound to CXCR4 can interfere with the “lock and key” mechanism between CXCR4 and CXCL12, which can result in inhibiting key functional signaling such as Matrigel invasion and cAMP modulation without displacing CXCL12.

### Secondary Function Assays

**Figure 2:**
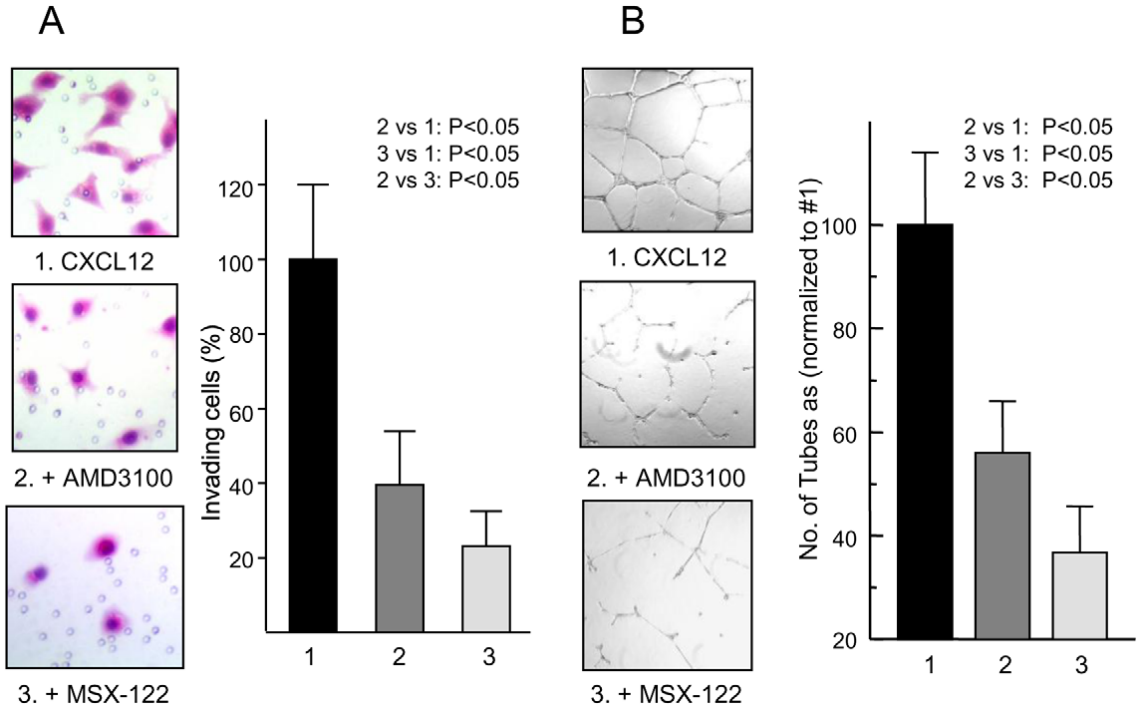
MSX-122 blocks invasion and angiogenesis *in vitro*. (A) Micrographs of Matrigel invasion assay induced by CXCR4/CXCL12-mediated interaction using MDA-MB-231 cells in the presence of anti-CXCR4 compounds. (B) Representative micrographs of the endothelial tubular network formation in the presence of anti-CXCR4 compounds. HUVECs formed excellent tubular networks in the presence of CXCL12 (#1), but poor tubular networks when CXCL12 was blocked by prior treatment with 100 nM of AMD3100 (#2) or MSX-122 (#3). The results are summarized in a graph in the right panel.

For secondary screening assays, we utilized three functional assays that use the full-length CXCL12 ligand. We previously reported that TN14003 effectively blocks CXCL12-mediated invasion of MDA-MB-231 cells in a Matrigel invasion assay using CXCL12 as a chemoattractant [20]. As shown in **Figure 2A**, 100 nM MSX-122 effectively blocked invasion of 78% MDA-MB-231 cells while the same concentration of AMD3100 blocked invasion of 62% cells. CXCR4 and CXCL12 are also known to play critical roles in endothelial cell migration and tubular organization. While AMD3100 at 100 nM blocked 43% of CXCL12-induced tubular network formation, MSX-122 blocked 63% at the same concentration (**Figure 2B**).

**Figure 3:**
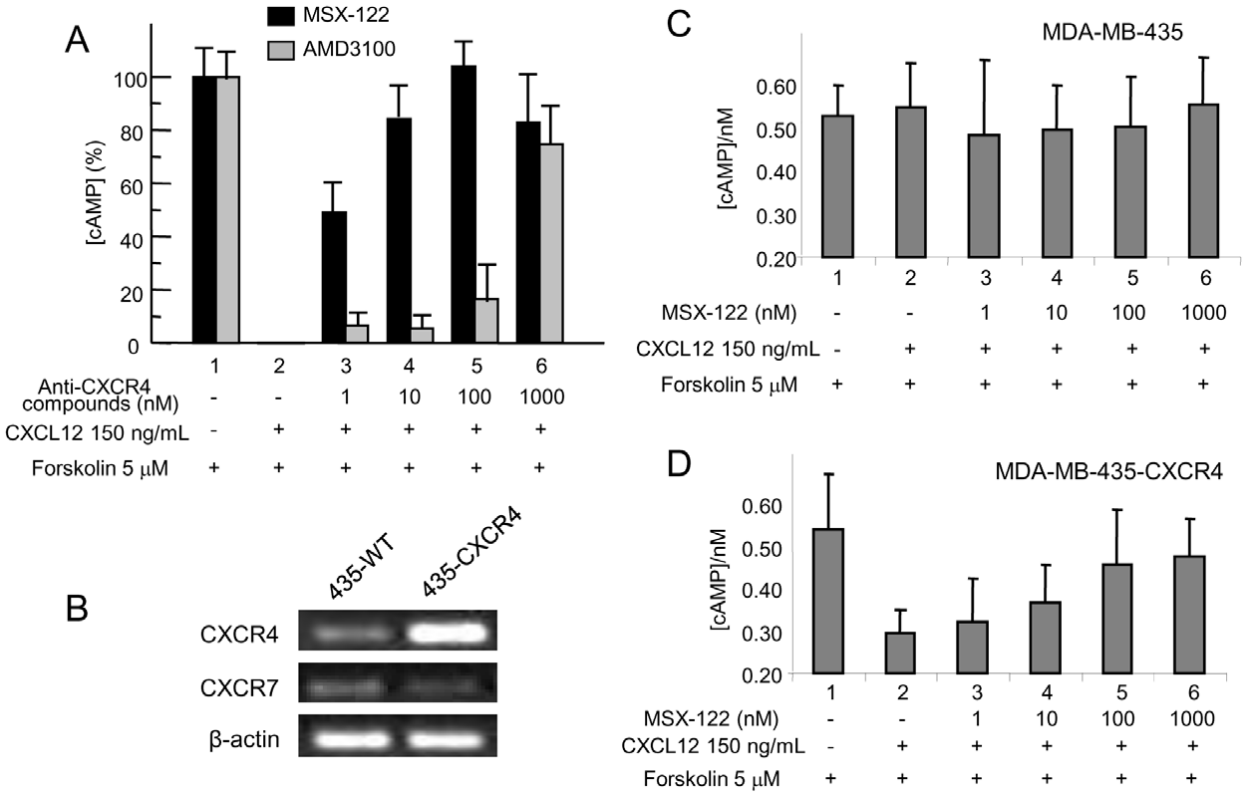
Comparison of inhibition of cAMP modulation by MSX-122 and AMD3100. (A) With 15 min pre-treatment with MSX-122 or AMD3100 at various concentrations, the effect of 150 ng/ml of CXCL12 on cAMP reduction was measured by the TR-FRET based LANCE assay kit using U87CD4CXCR4 cells. (B) RT-PCR analysis of CXCR4 and CXCR7 mRNA in wild-type MDA-MB-435 cells. CXCR4-overexpressing MDA-MB-435 cells were generated by transfection using a CXCR4 expressing plasmid (Missouri S&T cDNA resource center) and Lipofectamine2000. (C) No effect of MSX-122 on cAMP reduction induced by 150 ng/ml of CXCL12 in CXCR4-negative, wild-type MDA-MB-435 cells. (D) The effect of MSX-122 on cAMP reduction induced by 150 ng/ml of CXCL12 in CXCR4-overexpressing MDA-MB-435 cells.

Lastly, we utilized a cAMP assay as a secondary functional assay [23]. As a GPCR, CXCR4 binds CXCL12 and activates G-protein mediated signaling through the Gα_i_ pathway that reduces cAMP levels within cells. While MSX-122 counteracted CXCL12 function effectively at concentrations as low as 10 nM, 1000 nM AMD3100 was required to significantly block CXCL12 function (**Figure 3A**). We also determined whether CXCL12 alters cAMP levels in CXCR4-negative (wild-type) vs. CXCR4-transfected MDA-MB-435 cells to ensure that the cAMP changes in this assay are CXCR4-specific. It is noteworthy that neither MDA-MB-435 cells nor CXCR4-transfected MDA-MB-435 cells express CXCR7 (**Figure 3B**). As anticipated, cAMP levels were not influenced by CXCL12 in CXCR4-negative, wild-type MDA-MB-435 cells (**Figure 3C**), in contrast to CXCR4-transfected MDA-MB-435 cells (**Figure 3D**). MSX-122 was also tested against other chemokine receptors (e.g., CCR3 and CCR5) for cross-reactivity, but it did not block cAMP reduction mediated by their corresponding ligands CCR3/CCL5 and CCR5/CCL5 (data not shown).

Two other *in vitro* assays demonstrated that MSX-122 exhibits a different profile than AMD3100 and other reported anti-CXCR4 compounds: (1) MSX-122 did not inhibit T-tropic HIV infection (via the formation of the CXCR4/CD4/GP120 complex), while AMD3100 did [17]; and (2) unlike AMD3100, MSX-122 proved to be inactive in our calcium flux assay.

### Anti-inflammatory Efficacy of MSX-122 in Three Different Animal Models

#### Murine experimental colitis model

**Figure 4:**
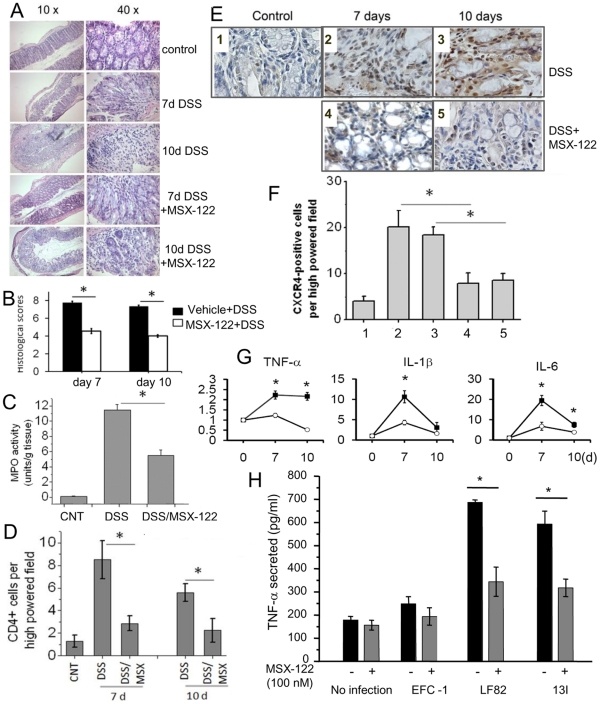
MSX-122 attenuates colonic damage in mice with experimental colitis. (A) Colonic sections were stained with H&E. (B) Histological scores were determined by a blinded examination of the sections. n = 16 per group. *, p<0.05 between DSS and DSS + MSX-122 treated mice. (C) MPO activity was measured in the colons of control, DSS-, and DSS/MSX-122-treated mice. MPO activity was expressed as unit of MPO per mg colonic lysate. n = 12. *, p<0.05. (D) The presence of CD4^+^ lymphocytes was detemined by immunohistochemically using anti-CD4 antibodies. CD4^+^ lymphocytes are depicted in the brown color and nuclei were counter-stained in the blue color. Quantification of CD4^+^ lymphocyte per high powered field is shown. n = 4–5. *, p<0.01. (E) Representatives of immnohistological staining of CXCR4-positive cells are shown. CXCR4 protein is depicted by the brown color, whereas nuclei were counter-stained in blue. 1, control; 2, 7 days DSS; 3, 7 days DSS + 3 days or recovery; 4, 7 days DSS/MSX-122; and 5, 7 days DSS/MSX-122 + 3 days MSX-122. (F) Quantitative analysis of CXCR4-positive immune cells in the colon is shown. *, p<0.01. MSX-122 blocks migration of CXCR4 positive cells. (G) Expression of cytokine mRNAs was determined by qRT-PCR. Expression levels of cytokines were normalized to 18S. n = 8. *, p<0.05. (H) MSX-122 attenuates TNF-α secretion induced by invasive *E.coli*. J774A.1 macrophages in triplicates were infected with overnight cultures of commensal *E.coli* EFC-1, CD-isolated *E.coli* LF82 or 13I at MOI 10∶1 for 3 h. Following 3 h infection, macrophage culture was washed and incubated for additional 21 h in a growth medium containing antibiotics and MSX-122. The amounts of TNF-α in the supernatant were quantified by ELISA. Representative data from three independent experiments, each performed in triplicate, are shown. *, p<0.05.

We sought to determine the therapeutic potential of MSX-122 by determining its profile in several *in vivo* assays associated with anti-CXCR4 properties. Previously, a TN14003 peptide analog and AMD3100 have been shown to block murine experimental colitis [39], [40]. To determine the efficacy of MSX-122 in this model, mice were treated with dextran sulfate sodium (DSS) for 7 days, followed by a 3 day recovery with or without MSX-122. DSS treatment resulted in inflammation manifested by extensive mucosal damage, epithelial erosion, crypt destruction, and infiltration of inflammatory cells in the mucosa and the lamina propria (**Figure 4A**). DSS/MSX-122-treated mice also developed signs of inflammation, but the degree of epithelial erosion and crypt destruction was significantly less, consistent with the clinical assessment, which quantifies the appearance of occult blood and diarrhea (data not shown). At day 10, colonic damage with crypt destruction and increased inflammatory cell infiltration remained in DSS-treated mice. By contrast, the colons of DSS/MSX-122-treated mice showed signs of recovery. **Figure 4B** quantitiates these histological features.

Consistent with the histological differences, a marked decrease in neutrophil infiltration into the injured tissue (as assessed by myeloperoxidase (MPO) activity [41]) was observed in DSS/MSX-122 treated mice when compared with DSS treated mice (**Figure 4C**). To elucidate whether MSX-122 action inhibits T-lymphocyte infiltration in colonic mucosa, we determined the number of CD4^+^ T-cells by immunohistochemical analysis [42] and found it to be markedly decreased in DSS/MSX-122-treated mice relative to DSS-treated mice (**Figure 4D**). These data clearly demonstrate that inflammation was far less severe in MSX-122-treated *vs.* DSS-treated mice.

Since inflammatory cells, including lymphocytes, neutrophils, and macrophages, express CXCR4, we wanted to determine whether MSX-122 blocks accumulation of such cells in the mucosa. DSS-treatment for 7 days significantly increased the number of CXCR4-positive cells at the sites of ulceration, which remained elevated at day 10. By comparison, the number of CXCR4-positive cells was markedly decreased in DSS/MSX122 treated animals relative to the DSS-treated group (**Figures 4E, F**). These data show that MSX-122 blocks infiltration of CXCR4-positive cells in the mucosa.

Since cytokines play a central role as mediators of inflammation, we next sought to determine the role of CXCR4 in the production of pro-inflammatory cytokines in the colon of mice by real-time RT-PCR. In DSS-treated mice, drastic increases in the mRNA levels of several pro-inflammatory cytokines were observed. MSX-122 attenuated the production of TNF-α, IL-1β, and IL-6 at day 7, and further decreased cytokine concentration at day 10 (**Figure 4G**). INF-γ mRNA expression was slightly reduced by MSX-122 treatment, but did not reach a statistical difference compared with DSS-treated mice (data not shown). As a consequence, we conclude that the inhibtion of CXCR4 alters the cytokine profiles within the mucosa of DSS-treated mice.

There is substantial evidence that inflammatory bowel disease (IBD), especially Crohn’s disease (CD), is associated with invasive *E.coli*, which regulates cytokine expression and epithelial barrier function within the host environment [43]. To demonstrate that MSX-122 attenuates the level of TNF-α, a critical inflammatory cytokine, we assessed TNF-α secretion by J774A.1 macrophages infected with invasive *E coli* isolated from inflamed mucosa of patients with CD, LF82, and 13I, or non-pathogenic EFC-1 as a control [43], [44]. Macrophages were incubated with bacterial culture in the absence or presence of MSX-122. The CD isolates, LF82 and 13I, greatly induced TNF-α secretion by macrophages [43]. The addition of MSX-122 to the culture media during the bacterial infection significantly attenuated the amount of TNF-α induced by these strains (**Figure 4H**), suggesting that CXCR4 plays crucial roles in the host response to the CD-isolated invasive *E.coli*.

#### Carrageenan-induced paw edema model

**Figure 5:**
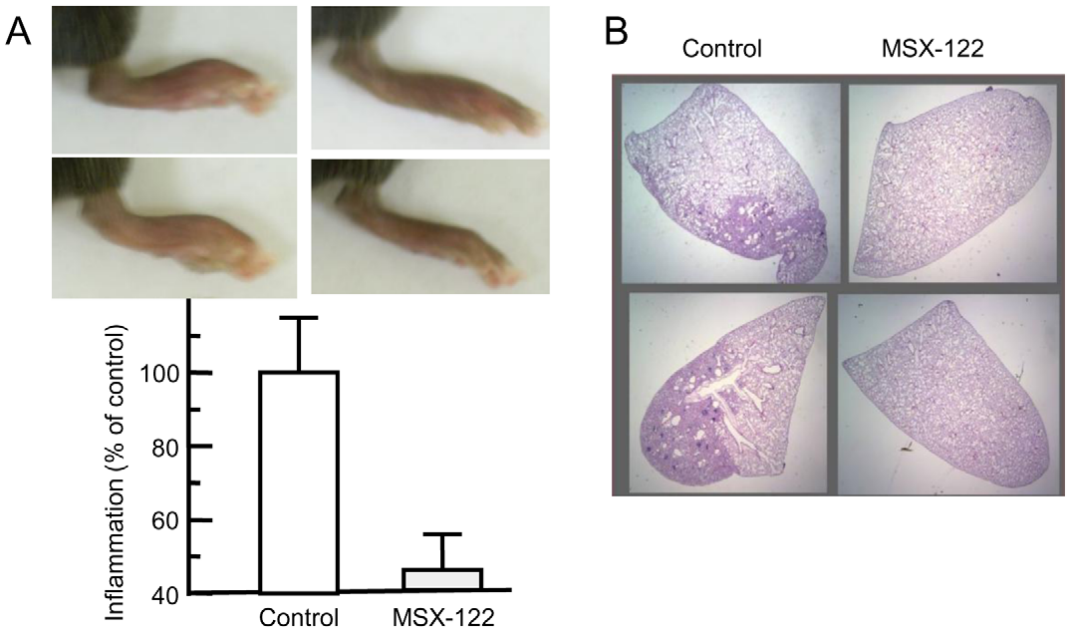
MSX-122 inhibits inflammation induced by carrageenan and lung fibrosis induced by bleomycin. (A) Suppression effect of anti-CXCR4 compounds on carrageenan-induced mouse paw edema. Acute paw inflammation was induced by subcutaneous injection of 50 µL of λ-carrageenan in one hind paw. The mice in the treatment group were all administered CXCR4 antagonists at 10 mg/kg i.p., while control animals received corresponding i.p. injections of vehicle. Left panels show the control mice with left paw induced inflammation by carrageenan; right panels show the treated mice with left paw induced inflammation by carrageenan with about 55% suppression. (B) MSX-122 inhibits bleomycin-induced pulmonary fibrosis. A total of 20 mice, 10 in each group, received 10 mg/kg, i.p. of MSX-122ms or saline one day before bleomycin treatment and daily for 20 days. Representative H&E stainings of saline treated control (left) or MSX-122ms treated (right) lung tissues on day 20 after bleomycin treatment.

Previously, we reported utilizing a carrageenan-induced mouse paw edema model as one of our screening cascades for anti-CXCR4 compounds [45]. It is a widely used test to assess anti-inflammatory activity *in vivo*, which is known to involve CXCR4 as a key role in the recruitment of inflammatory cells to sites of inflammation. An apparent edema response was seen 24 h after the λ-carrageenan injection (compared to the contralateral paw that was injected with saline). **Figure 5A** shows that MSX-122 exhibits more than 50% inhibitory effect on inflammation. These data confirm that MSX-122 can inhibit inflammation as anticipated.

#### Bleomycin-induced lung fibrosis model

Previously, we had studied the role of the CXCL12/CXCR4-axis in a rodent model of bleomycin-induced lung injury and reported that TN14003 blocked bleomycin-induced lung fibrosis [22]. Using the same model, we tested whether MSX-122 blocks bleomycin-induced lung fibrosis. Lungs harvested 20 days after bleomycin treatment were analyzed histologically. None of the MSX-122ms (salt form was used for better solubility)-treated mice exhibited lung fibrosis, while bleomycin caused marked increases in collagen deposition in the control mice (**Figure 5B**). Thus, treatment with MSX-122ms completely prevented bleomycin-induced lung fibrosis, demonstrating superiority over TN14003 [22].

### Anti-metastatic Efficacy of MSX-122 in Three Different Animal Models

**Figure 6:**
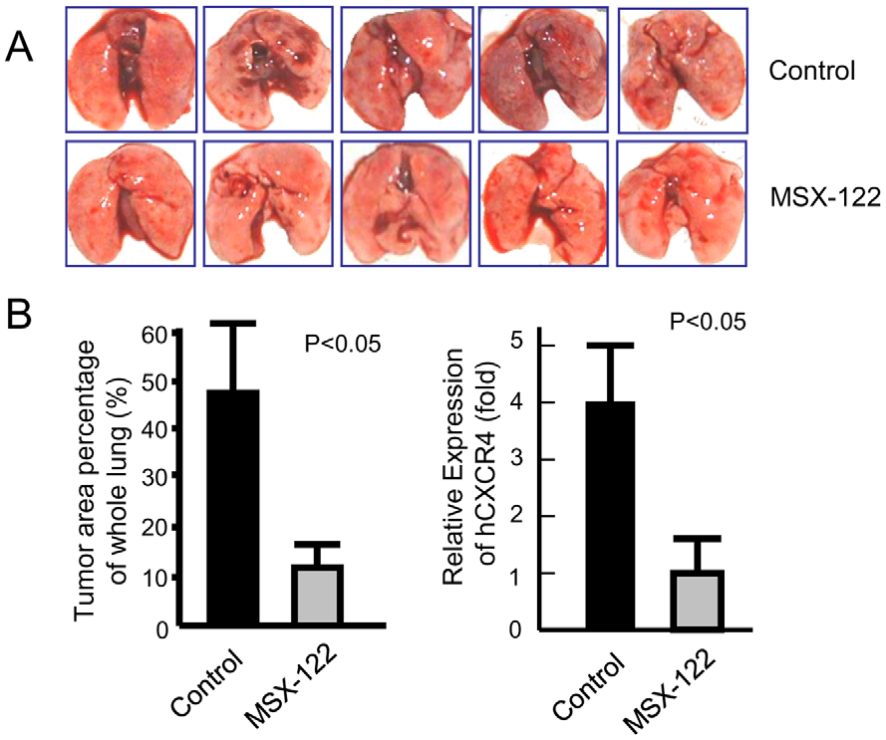
MSX-122 blocks metastasis in an experimental animal model of breast cancer metastasis. (A) Representative photographs of 5 lungs were shown from each group. (B) Histological quantization shows a decrease in tumor area in the treated group. Quantitative real-time RT-PCR of human CXCR4 confirms that animals injected with MDA-MB-231 cells and treated with vehicle develop lung metastasis. On the other hand, animals injected with MDA-MB-231 cells and treated with MSX-122 (4 mg/kg, i.p., daily) develop significantly less metastasis.

To evaluate the anti-metastatic efficacy of MSX-122, we utilized three different animal models. For breast cancer metastasis, MDA-MB-231 cells were injected intravenously to generate an experimental metastasis model. All untreated control mice developed lung metastases (**Figure 6A** **top**), while the group treated with MSX-122ms, i.p. exhibited significantly fewer lung metastases (**Figure 6A** **bottom**). The estimated average areas of micrometastases on the lung surface from the control and treated groups were 47.5% and 13%, respectively (**Figure 6B** **left**) as confirmed by real-time RT-PCR using primers to detect human CXCR4 (**Figure 6B** **right**).

**Figure 7:**
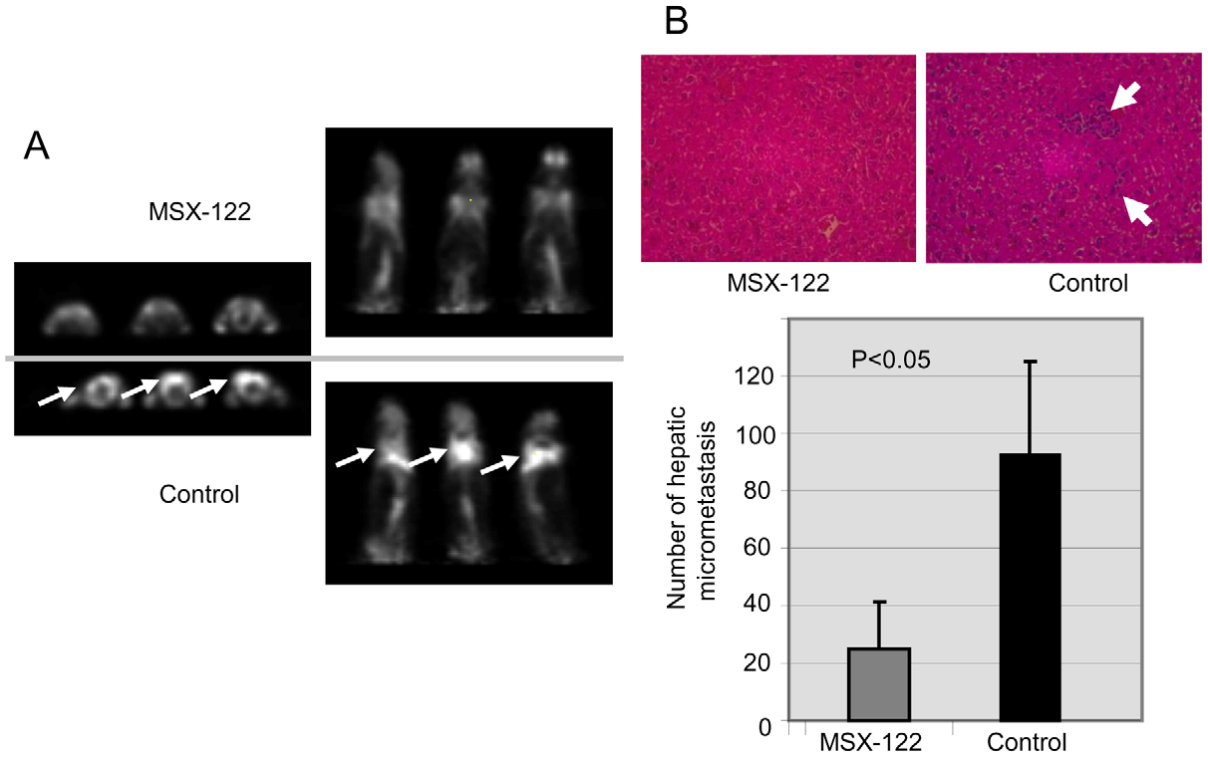
MSX-122 blocks metastasis in two more animal models for metastasis. (A) Non-invasive [^18^F]FDG-PET imaging of lung metastasis in head and neck cancer animal models. Three randomly selected mice revealed lung metastases in all mice in the control group; significantly less metastasis was found in the MSX-122 (10 mg/kg i.p., daily) treated group. Left panels show the axial images of three subjects from the control group (bottom) and three from the treated group (top). Right panels show the corresponding coronal images of the same mice. White arrows indicate metastasized SCCHN tumor cells. (B) The inhibition effect of MSX-122 (10 mg/kg i.p., daily) in a uveal melanoma micrometastasis animal model. Micrometastatic clones in liver are shown by small white arrows in the images. MSX-122 significantly decreased the numbers of hepatic micrometastases.

The anti-metastatic efficacy of MSX-122ms was confirmed in a model for SCCHN metastasis known for the critical role of CXCR4 in metastatic progression using [^18^F]FDG-PET which is a standard imaging tool to detect lung metastasis in clinic [21]. In **Figure 7A** (left panel), [^18^F]FDG-PET axial images of three randomly selected mice from each group (6 mice in one scan) are shown (the metastasized SCCHN tumor cells are indicated by white arrows). When injected with control vehicle, the mice exhibited significant lung metastases (bottom three mice). By contrast, the arm administered with MSX-122ms showed no evidence of metastases, similar to TN14003 [21]. The two panels on the right (**Figure 7A**) are the coronal images of the same mice on the left. The top three mice were treated with MSX-122ms, while the bottom three mice represent the control.

To further test whether MSX-122 can also inhibit metastatic progression in an orthotopic mouse model of uveal melanoma micrometastasis [46], [47], melanoma OMM2.3 cells overexpressing HGF/TGF-β/CXCR4/MMP2 were inoculated into the posterior chamber of the right eye. After 3 days, MSX-122 treatment was initiated, and after one week, the right eye that developed tumor was enucleated. After 4 weeks, the mice were sacrificed and hepatic tissues were collected and processed for H&E staining (**Figure 7B**). Six sections through the center of the liver were microscopically examined for the presence of micrometastases, and the total number of micrometastases in the treatment group was determined to be 24 (**Figure 7B** **, bar graph**). This was significantly less than the control group at 93 (p<0.05) and TN14003 at 40 [45].

## Discussion

Metastasis is an end result for many solid tumor types and is the leading cause of cancer related deaths. This process involves intimate cooperation between tumor cells and their tumor microenvironment for homing and recruitment of tumor cells as well as tumor stroma components. Ironically, as of now, there are no marketed therapies that specifically target and address the metastatic process. As such, it has attracted an increasing amount of attention from research groups over the last decade in both academia and industry, as a target for disease intervention and life extension. The CXCR4/CXCL12 interaction, and the resulting cell signaling cascade, has recently emerged as one of the most relevant such targets as it has been shown to play a key role in metastatic progression. We have been simultaneously designing and generating new analogs to optimize their efficacy as well as expand our structural scope [23], [45]. MSX-122 is currently our most advanced lead, which potently binds to CXCR4, thus, blocking certain functions of CXCR4/CXCL12 signaling. We have followed this discovery by demonstrating that MSX-122 inhibits tumor metastasis and inflammation in numerous *in vitro* and *in vivo* studies. Specific screening assays that have been established for the selection process include *in vitro* binding potency, ligand competition, Matrigel invasion, and cAMP modulation, and CXCR4/MSX-122 docking modeling. We have also established and validated a number of *in vivo* models of cancer metastasis and inflammation that have been utilized here to establish a proof of efficacy for our lead molecule. The intended outcome of current work was the development of a unique small molecule that will effectively attenuate cancer metastasis *in vivo* by blocking CXCR4 function whilst demonstrating a specificity profile to merit advancement into human clinical evaluation.

The anti-inflammatory efficacy of MSX-122 has been tested in three animal models in which inhibition of CXCR4 is reported to ameliorate the severity of disease progression. We observed that DSS/MSX-122-treated mice had shown only moderate signs of inflammation compared to DSS-treated mice. In addition, we demonstrated that MSX-122 blocks bleomycin-induced lung fibrosis involving chemotaxis and homing of CXCR4-positive mesenchymal progenitor cells into the lungs, the same critical mechanisms involved in cancer metastasis. Furthermore, MSX-122 exhibits anti-inflammatory activity in a carrageenan-induced paw edema model. Consistent with the effect of other known CXCR4 antagonists, MSX-122 blocks lung metastasis of breast cancer and SCCHN, and liver metastasis of uveal melanoma *in vivo*. These data further support the role of CXCR4 in chemotaxis, motility, and invasion and reinforce its value as a target for therapeutic intervention in disease states where these phenomena play key roles, such as inflammation and cancer metastasis.

A most intriguing aspect of MSX-122 is that it behaves differently from other reported anti-CXCR4 compounds, especially AMD3100. MSX-122 is not effective in blocking T-tropic HIV infections as determined by studies carried out at Monogram Biosciences (data not shown). MSX-122 is also incapable of blocking the binding of ^125^I-labeled CXCL12 to CXCR4. Consistent with previous reports of multiple interaction sites between CXCR4 and CXCL12 [27], MSX-122 (molecular weight <300 daltons) appears to be insufficiently large to block all binding sites between CXCR4 and CXCL12. However, we anticipated that CXCL12 would block MSX-122 binding to CXCR4 because MSX-122 binds to a CXCL12 binding site on CXCR4. To test this hypothesis, we labeled MSX-122 with fluorine-18 and tested whether preincubation with CXCL12 before adding [^18^F]MSX-122F blocked binding to CXCR4 in the CXCR4-positive metastatic SCCHN cells. As predicted, CXCL12 blocked binding of [^18^F]MSX-122F to CXCR4. This strongly supports that MSX-122 blocks certain CXCR4 functions via binding to the CXCL12-binding site and interfering with CXCR4/CXCL12-mediated signaling.

Like our previous anti-CXCR4 compounds, MSX-122 can intervene in the Gα_i_-signaling pathway (cAMP modulation), but not the Gq-pathway (calcium flux) [48]. This limited functionality of MSX-122 as a CXCR4 inhibitor may represent a significant safety asset, since the CXCL12/CXCR4-axis is critical to normal physiology. In the normal adult, the CXCL12/CXCR4 signaling is involved in the homing and retention of hematopoietic progenitor cells in the bone marrow. These progenitor cells express high levels of CXCR4, and are attracted to CXCL12 produced by stromal cells in specialized bone marrow niches. In addition, CXCL12 acts as a major chemoattractant for stem cells and some differentiated cells in the pathological contexts of tissue regeneration/repair [49], [50], [51], [52]. Therefore, our functionally circumscribed CXCR4 inhibitor, which blocks chemotaxis and homing of the CXCR4-positive cells to distant organ sites when enriched with CXCL12 in the stroma without disturbing the retention of hematopoietic progenitor cells in the bone marrow, presages a unique therapeutic application of MSX-122 for cancer metastasis or inflammation.

In summary, the novel small molecule, MSX-122, identified by rational design and analysis of emerging structural and pharmacologic data, is a partial inhibitor of CXCR4/CXCL12 functions. The compelling features of the MSX-122 profile include: (i) potent inhibition of CXCR4/CXCL12 actions (IC_50_∼10 nM); (ii) ease of production on a large scale; (iii) effectiveness as an anti-inflammatory and anti-metastatic agent *in vivo*; and (iv) unique property of blocking homing and recruitment without mobilizing stem cells.

## Materials and Methods

### Synthesis of N,N'-Di-2-pyrimidinyl-1,4-benzenedimethanamine (MSX-122)

MSX-122 was prepared similarly to the preparation of WZ811 [23] following a one-pot reductive amination strategy (**Figure 1A**) [24]. Details of MSX-122 synthesis and characterization data are provided in Data S1.

### A Binding Affinity Assay, Invasion Assay, and cAMP Assay

For binding affinity assay, MDA-MB-231 cells cultured in an 8-well slide chamber were preincubated with MSX-122 at 1, 10, 100, and 1000 nM. Then the cells were fixed with 4% formaldehyde and incubated with 50 nM biotinylated TN14003, and followed by Rhodamine staining [23]. Matrigel invasion chambers from BD Biocoat Cellware (San Jose, CA) were used for invasion assays. MDA-MB-231 cells were cultured on a layer of Matrigel in the upper chamber with testing compounds at 10 or 100 nM while 200 ng/mL CXCL12 was added in the lower chamber as a chemoattractant. cAMP assay was performed using U87CD4CXCR4 cells (AIDS Consortium) or MDA-MB-435 cells transfected with CXCR4 plasmid (Missouri S&T cDNA resource center). Perkin-Elmer’s LANCE cAMP assay kit (Cat # AD0262), based on time-resolved fluorescence resonance energy transfer (TR-FRET), was used to determine whether MSX-122 could block cAMP modulation induced by the CXCR4/CXCL12 interaction as previously described [23].

### F-18 Labeling

Fluorine-18 was prepared by the ^18^O (p,n) ^18^F reaction using ^18^O enriched water by PetNet Solutions at Emory University Yerkes National Primate Center. The aqueous solution was transferred into a vial located in a hot cell in the Yerkes Radiochemistry Laboratory. The solution was then transferred into a mini cell which houses a chemical process control unit where ^18^F was trapped on an anion exchange resin cartridge. The ^18^F was then eluted with a potassium carbonate solution into a vessel containing Kryptofix 2,2,2 and the mixture dried by azeotropic distillation with acetonitrile. The complexed ^18^F was reacted with 3 mg chloro- precursor in DMSO at 170°C. The diluted reaction product was purified by C_18_ reverse phase HPLC using a Waters Xterra Prep RP18 5 µm, 19×100 mm column and a mobile phase of 50∶50∶0.1 EtOH: H_2_O: Et_3_N. The desired compound was isolated from the diluted fractions by C_18_ solid phase extraction and eluted by ethanol into a vial containing isotonic saline. The mixture was sterilized by filtration through a 0.2 micron filter. The chemical and radiochemical purity was determined by analytical HPLC utilizing a Waters Nova-Pak C_18_ 3.9×150 mm column with a mobile phase of 70∶30∶0.1 MeOH: H_2_O: Et_3_N. The specific activity was estimated based upon the lower limit of detectable unlabeled reference compound.

### Ligand Competition

The cell binding assays were performed with SCCHN metastatic subclones 686LN-Ms cells. In this study, approximately 2×10^6^ cells were exposed to 5 µCi of F-18 labeled radioligand in 1% BSA in PBS (actual MSX-122F concentration was 12.5 nM) and incubated at 37°C for 60 minutes. Each assay condition was performed in triplicate. For competition test, the cells were preincubated with different concentrations of CXCL12 of 11, 33, 100 and 400 nM for 15 mins, and then incubated with the radioligands in the same condition as above. After incubation, cells were centrifuged and rinsed with ice-cold 1% BSA in PBS to remove unbound activity in the supernatant. The activity in the tubes was counted in an automated γ-counter (AccuFLEXγ7001, Aloka, Co. Ltd., Japan). The data from these studies were normalized as percent uptake relative to standard per 2×10^6^ cells.

### Real-time RT–PCR Analysis

Human CXCR4-specific primers and ß-actin primers used were the same as those described in our previous report [20]. The human CXCR7-specific primers for 220 bp are 5′-ACGTGGTGGTCTTCCTTGTC and 5′-AAGGCCTTCATCAGCTCGTA (GenBank accession number NM_020311). The primers for TNF-α are 5′-AGGCTGCCCCGACTACGT and 5′-GACTTTCTCCTGGTATGAGATAGCAAA (GenBank accession number NM_013693), those for IL-1β are 5′- TCGCTCAGGGTCACAAGAAA and 5′- CATCAGAGGCAAGGAGGAAAAC (GenBank assession number NM_008361), and those for IL-6 are 5′-ACAAGTCGGAGGCTTAATTACACAT and 5′-TTGCCATTGCACAACTCTTTTC (Genbank accession number X54542). Details are provided in Table S1.

Detailed conditions and data analysis were described in our previous study [53].

### DSS-induced Colitis

Male C57BL/6 mice (age 5–6 weeks) were treated with 3% DSS in drinking water to induce colitis. To determine the effect of MSX-122, a group of mice were given DSS and daily i.p. injection of MSX-122, 10 mg/kg (dissolved in 10% DMSO and 45% 2-hydroxypropyl-β cyclodextrin (Fluka)). Half of the DSS-treated mice were sacrificed at day 7, whereas the remaining mice were kept for an additional 3 days. Control mice were given either daily i.p. injection of vehicle or MSX-122 for 10 days. The colon was removed and processed as previously reported [54]. Neutrophil infiltration was quantified by measuring myeloperoxidase (MPO) activity as described previously [41]. Anti-CXCR4 antibody and anti-CD4 antibody were purchased from Abcam and BD Pharmingen, respectively.

### Bleomycin-induced Lung Fibrosis

Mice were anesthetized by isofluorane inhalation; the trachea was exposed using sterile techniques and 4 U/kg bleomycin (Sigma) in 100 µL PBS or PBS vehicle was injected into the tracheal lumen. After inoculation, the incision was closed and the animals were allowed to recover. Each group has 10 mice that received 10 mg/kg of MSX-122ms or PBS intraperitoneally 1 day before bleomycin treatment and daily for 20 days. Lungs harvested 20 days after bleomycin treatment were analyzed histologically by H&E-staining and imaged under microscope [45].

### Paw Inflammation Suppression Test

Acute inflammation was induced by subcutaneous injection of 50 µL of λ-carrageenan (1% w/v in saline) into one of the hind paws of female C57BL/6J mice (Jackson Laboratories); the other hind paw was used as a non-inflammation control. In the treatment group, MSX-122 was administered i.p. at 10 mg/kg, 30 min following carrageenan challenge and continued daily. The animals were sacrificed 74 h after induction of inflammation and 2 h after the last injection of MSX-122. The final paws were photographed and measured for thickness from the “palm” to the back of the paw by a caliper. These were compared to the volume of carrageenan untreated contralateral paw to obtain the edema volume; the volume of the contralateral paw was subtracted from the volume of the carrageenan injected paw to obtain the edema volume. The inflammation suppression percentage was calculated by comparing the drug treated group to the control group. Twelve mice per group were used to determine the effect of MSX-122 on acute inflammation as previously described [45].

### Animal Experiments for Metastasis

Six- to eight-week-old female nude mice (Taconic Farms) were given injections of 1.5×10^6^ MDA-MB-231 breast cancer cells mixed with the compound (1 µM, less than 5 min preincubation) through the tail vein (10/group). From the following day, mice in the treated group were given 4 mg/kg MSX-122ms daily by i.p. injection. The animals were sacrificed 35 days after the tumor cell injection. Whole lung tissues were harvested and sectioned for real-time RT-PCR for human CXCR4 and H&E histostaining to evaluate the metastatic tumor area in five fields per section microscopically. These experiments were repeated once more to confirm the results. For the head and neck cancer animal model, metastatic subclones of 686LN-Ms cells were injected in the same way as MDA-MB-231 cells as described previously [21]. [^18^F]FDG-PET was performed as described previously [21]. For the uveal melanoma micrometastasis mouse model, on day 0, each mouse was inoculated with 1×10^6^ wild-type OMM2.3 cells expressing HGF/TGF-β/CXCR4/MMP2 into the posterior chamber of right eye. On day 3, mice were treated with 10 mg/kg MSX-122 in 0.1 mL volume of 45% CD daily by i.p. injection, whereas the control mice were injected with 0.1 mL 45% CD only. On day 7, eyes with tumor were enucleated. The growth of tumor was checked by histological methods. On day 28, hepatic tissues were collected and fixed in 10% formalin, processed, H&E stained, and the number of hepatic micrometastases was counted under microscope. Six sections through the center of the liver were microscopically examined (Olympus BX41, Tokyo, Japan) for the presence of micrometastases (<100 µm diameter) and the average number of micrometastases per section was determined [45]. Ten mice per group were used. A table summarizing animal experiments for three metastasis models can be found in the Data S3.

All the animals were housed in the Emory animal facility. All protocols for animal studies were reviewed and approved by the Institutional Animal Care and Use Committee at Emory University ( IACUC protocol numbers:170-2008, 155-2007, 029-2008, and 065-2006). Emory DAR (Division of Animal Resources) has experienced clinical veterinary and animal caretaker support staff who are well trained in the care of rodents. All animals were monitored daily by the investigators and by members of the clinical veterinary staff concerning general health and to detect signs of discomfort. Any animal showing signs of discomfort, pain or distress was sacrificed immediately following IACUC guideline for endpoints. All reasonable measures were taken to ensure the health and well being of the animals during the course of the study. The procedures to be used for animal care were consistent with those established by the NIH and the vivarium was fully accredited by the AAALAC. All experimental results adhere to the ARRIVE guidelines for the reporting of animal research [55].

### Statistical Analyses

All statistical significances were determined by Student’s *t-*test.

## Supporting Information

Table S1 **Primer sequences.**
(DOCX)

Data S1 **Synthesis and characterization of MSX-122.**
(DOCX)

Data S2 **Structure of various anti-CXCR4 compounds.**
(DOCX)

Data S3 **Summary of animal experiments for three metastasis models.**
(DOCX)

## References

[1] Davis CB, Dikic I, Unutmaz D, Hill CM, Arthos J (1997). Signal transduction due to HIV-1 envelope interactions with chemokine receptors CXCR4 or CCR5.. J Exp Med.

[2] Feng Y, Broder CC, Kennedy PE, Berger EA (1996). HIV-1 entry cofactor: functional cDNA cloning of a seven-transmembrane, G protein-coupled receptor.. Science.

[3] Sanchez X, Cousins-Hodges B, Aguilar T, Gosselink P, Lu Z (1997). Activation of HIV-1 coreceptor (CXCR4) mediates myelosuppression.. J Biol Chem.

[4] Zaitseva M, Blauvelt A, Lee S, Lapham CK, Klaus-Kovtun V (1997). Expression and function of CCR5 and CXCR4 on human Langerhans cells and macrophages: implications for HIV primary infection.. Nat Med.

[5] Calandra G, Bridger G, Fricker S (2010). CXCR4 in clinical hematology.. Curr Top Microbiol Immunol.

[6] Zlotnik A (2006). Involvement of chemokine receptors in organ-specific metastasis.. Contrib Microbiol.

[7] Liotta LA, Kohn EC (2001). The microenvironment of the tumour-host interface.. Nature.

[8] Porta C, Riboldi E, Sica A (2010). Mechanisms linking pathogens-associated inflammation and cancer..

[9] Lazennec G, Richmond A (2010). Chemokines and chemokine receptors: new insights into cancer-related inflammation.. Trends Mol Med.

[10] Kucia M, Reca R, Miekus K, Wanzeck J, Wojakowski W (2005). Trafficking of normal stem cells and metastasis of cancer stem cells involve similar mechanisms: pivotal role of the SDF-1-CXCR4 axis.. Stem Cells.

[11] Furusato B, Mohamed A, Uhlen M, Rhim JS (2010). CXCR4 and cancer.. Pathol Int.

[12] Weinberg RA (2007). The Biology of Cancer: Garland Science.

[13] Kang Y, Siegel PM, Shu W, Drobnjak M, Kakonen SM (2003). A multigenic program mediating breast cancer metastasis to bone.. Cancer Cell.

[14] Muller A, Homey B, Soto H, Ge N, Catron D (2001). Involvement of chemokine receptors in breast cancer metastasis.. Nature.

[15] Balkwill F (2004). The significance of cancer cell expression of the chemokine receptor CXCR4.. Semin Cancer Biol.

[16] Shim H, Oishi S, Fujii N (2009). Chemokine receptor CXCR4 as a therapeutic target for neuroectodermal tumors.. Semin Cancer Biol.

[17] De Clercq E (2003). The bicyclam AMD3100 story.. Nat Rev Drug Discov.

[18] Strieter RM, Keeley EC, Hughes MA, Burdick MD, Mehrad B (2009). The role of circulating mesenchymal progenitor cells (fibrocytes) in the pathogenesis of pulmonary fibrosis.. J Leukoc Biol.

[19] Schiffer D, Annovazzi L, Caldera V, Mellai M (2012). On the origin and growth of gliomas.. Anticancer Res.

[20] Liang Z, Wu T, Lou H, Yu X, Taichman RS (2004). Inhibition of breast cancer metastasis by selective synthetic polypeptide against CXCR4.. Cancer Res.

[21] Yoon Y, Liang Z, Zhang X, Choe M, Cho HT (2007). CXCR4 antagonist blocks both growth of primary tumor and metastasis of head and neck cancer in xenograft mouse models.. Cancer Res.

[22] Xu J, Mora A, Shim H, Stecenko A, Brigham KL (2007). Role of the SDF-1/CXCR4 Axis in the Pathogenesis of Lung Injury and Fibrosis..

[23] Zhan W, Liang Z, Zhu A, Kurtkaya S, Shim H (2007). Discovery of small molecule CXCR4 antagonists.. J Med Chem.

[24] Abdel-Magid AF, Carson KG, Harris BD, Maryanoff CA, Shah RD (1996). Reductive Amination of Aldehydes and Ketones with Sodium Triacetoxyborohydride. Studies on Direct and Indirect Reductive Amination Procedures(1).. J Org Chem.

[25] Thelen M, Thelen S (2008). CXCR7, CXCR4 and CXCL12: an eccentric trio?. J Neuroimmunol.

[26] Wang J, Shiozawa Y, Wang J, Wang Y, Jung Y (2008). The role of CXCR7/RDC1 as a chemokine receptor for CXCL12/SDF-1 in prostate cancer.. J Biol Chem.

[27] Gupta SK, Pillarisetti K, Thomas RA, Aiyar N (2001). Pharmacological evidence for complex and multiple site interaction of CXCR4 with SDF-1alpha: implications for development of selective CXCR4 antagonists.. Immunol Lett.

[28] Fricker SP, Anastassov V, Cox J, Darkes MC, Grujic O (2006). Characterization of the molecular pharmacology of AMD3100: a specific antagonist of the G-protein coupled chemokine receptor, CXCR4.. Biochem Pharmacol.

[29] Kalatskaya I, Berchiche YA, Gravel S, Limberg BJ, Rosenbaum JS (2009). AMD3100 is a CXCR7 ligand with allosteric agonist properties.. Mol Pharmacol.

[30] Kofuku Y, Yoshiura C, Ueda T, Terasawa H, Hirai T (2009). Structural basis of the interaction between chemokine stromal cell-derived factor-1/CXCL12 and its G-protein-coupled receptor CXCR4.. J Biol Chem.

[31] Crump MP, Gong JH, Loetscher P, Rajarathnam K, Amara A (1997). Solution structure and basis for functional activity of stromal cell-derived factor-1; dissociation of CXCR4 activation from binding and inhibition of HIV-1.. EMBO J.

[32] Wang J, Zhang X, Thomas SM, Grandis JR, Wells A (2005). Chemokine receptor 7 activates phosphoinositide-3 kinase-mediated invasive and prosurvival pathways in head and neck cancer cells independent of EGFR.. Oncogene.

[33] Wu B, Chien EY, Mol CD, Fenalti G, Liu W (2010). Structures of the CXCR4 chemokine GPCR with small-molecule and cyclic peptide antagonists.. Science.

[34] Friesner RA, Banks JL, Murphy RB, Halgren TA, Klicic JJ (2004). Glide: a new approach for rapid, accurate docking and scoring. 1. Method and assessment of docking accuracy.. J Med Chem.

[35] Halgren TA, Murphy RB, Friesner RA, Beard HS, Frye LL (2004). Glide: a new approach for rapid, accurate docking and scoring. 2. Enrichment factors in database screening.. J Med Chem.

[36] Sherman W, Day T, Jacobson MP, Friesner RA, Farid R (2006). Novel procedure for modeling ligand/receptor induced fit effects.. J Med Chem.

[37] Graves AP, Shivakumar DM, Boyce SE, Jacobson MP, Case DA (2008). Rescoring docking hit lists for model cavity sites: predictions and experimental testing.. J Mol Biol.

[38] Guimaraes CR, Cardozo M (2008). MM-GB/SA rescoring of docking poses in structure-based lead optimization.. J Chem Inf Model.

[39] Xia XM, Wang FY, Xu WA, Wang ZK, Liu J (2010). CXCR4 antagonist AMD3100 attenuates colonic damage in mice with experimental colitis.. World J Gastroenterol.

[40] Mikami S, Nakase H, Yamamoto S, Takeda Y, Yoshino T (2008). Blockade of CXCL12/CXCR4 axis ameliorates murine experimental colitis.. J Pharmacol Exp Ther.

[41] Castaneda FE, Walia B, Vijay-Kumar M, Patel NR, Roser S (2005). Targeted Deletion of Metalloproteinase 9 Attenuates Experimental Colitis in Mice: Central Role of Epithelial-Derived MMP.. Gastroenterology.

[42] Andres PG, Beck PL, Mizoguchi E, Mizoguchi A, Bhan AK (2000). Mice with a Selective Deletion of the CC Chemokine Receptors 5 or 2 Are Protected from Dextran Sodium Sulfate-Mediated Colitis: Lack of CC Chemokine Receptor 5 Expression Results in a NK1.1+ Lymphocyte-Associated Th2-Type Immune Response in the Intestine.. J Immunol.

[43] Sasaki T, Sasaki J, Sakai T, Takasuga S, Suzuki A (2007). The physiology of phosphoinositides.. Biol Pharm Bull.

[44] Boudeau J, Glasser A-L, Masseret E, Joly B, Darfeuille-Michaud A (1999). Invasive Ability of an Escherichia coli Strain Isolated from the Ileal Mucosa of a Patient with Crohn's Disease.. Infect Immun.

[45] Zhu A, Zhan W, Liang Z, Yoon Y, Yang H (2010). Dipyrimidine Amines: A novel class of chemokine receptor type 4 antagonists with high specifity.. J Med Chem.

[46] Yang H, Fang G, Huang X, Yu J, Hsieh CL (2008). In-vivo xenograft murine human uveal melanoma model develops hepatic micrometastases.. Melanoma Res.

[47] Yang H, Grossniklaus HE (2006). Combined immunologic and anti-angiogenic therapy reduces hepatic micrometastases in a murine ocular melanoma model.. Curr Eye Res.

[48] Dorsam RT, Gutkind JS (2007). G-protein-coupled receptors and cancer.. Nat Rev Cancer.

[49] Gao C, Li Y (2007). SDF-1 plays a key role in the repairing and remodeling process on rat allo-orthotopic abdominal aorta grafts.. Transplant Proc.

[50] Imitola J, Raddassi K, Park KI, Mueller FJ, Nieto M (2004). Directed migration of neural stem cells to sites of CNS injury by the stromal cell-derived factor 1alpha/CXC chemokine receptor 4 pathway.. Proc Natl Acad Sci U S A.

[51] Kajiyama H, Shibata K, Ino K, Nawa A, Mizutani S (2007). Possible involvement of SDF-1alpha/CXCR4-DPPIV axis in TGF-beta1-induced enhancement of migratory potential in human peritoneal mesothelial cells.. Cell Tissue Res.

[52] Moyer RA, Wendt MK, Johanesen PA, Turner JR, Dwinell MB (2007). Rho activation regulates CXCL12 chemokine stimulated actin rearrangement and restitution in model intestinal epithelia.. Lab Invest.

[53] Liang Z, Brooks J, Willard M, Liang K, Yoon Y (2007). CXCR4/CXCL12 axis promotes VEGF-mediated tumor angiogenesis through Akt signaling pathway.. Biochem Biophys Res Commun.

[54] Lin S, Yeruva S, He P, Singh AK, Zhang H (2010). Lysophosphatidic acid stimulates the intestinal brush border Na(+)/H(+) exchanger 3 and fluid absorption via LPA(5) and NHERF2.. Gastroenterology.

[55] Kilkenny C, Browne WJ, Cuthill IC, Emerson M, Altman DG (2010). Improving bioscience research reporting: the ARRIVE guidelines for reporting animal research.. PLoS Biol.

