# Supplementary material for: Development of a Unique Small Molecule Modulator of CXCR4
Source: PLoS One. 2012 Apr 2;7(4):e34038. doi: 10.1371/journal.pone.0034038 (PMC3317778; doi:10.1371/journal.pone.0034038)
Supplement: Table S1 — Primer sequences. (DOCX) [file pone.0034038.s001.docx]

**Table S1**

| **Genes** |  | **Sequences** | **Size of PCR products** |
| --- | --- | --- | --- |
| TNF-α | forward | aggctgccccgactacgt | 171 bp |
| (NM_013693) | reverse | gactttctcctggtatgagatagcaaa |  |
| IFN-γ | forward | cagcaacagcaaggcgaaa | 73 bp |
| (NM_008337) | reverse | ctggacctgtgggttgttgac |  |
| IL-1β | forward | tcgctcagggtcacaagaaa | 73 bp |
| (NM008361) | reverse | catcagaggcaaggaggaaaac |  |
| IL-6 | forward | acaagtcggaggcttaattacacat | 70 bp |
| (X54542) | reverse | ttgccattgcacaactcttttc |  |
| MCP-1 | forward | actgaagccagctctctcttcctc | 274 bp |
| (NM_011333) | reverse | ttccttcttggggtcagcacagac |  |
| CXCR4(mouse) | forward | tcagtggctgacctcctctt | 203 bp |
| (NM_009911) | reverse | cttggcctttgactgttggt |  |
| CXCR4(human) | forward | gaaccctgtttccgtgaaga | 151 bp |
| (NM_003467) | reverse | cttgtccgtcatgcttctca |  |
| SDF1(mouse) | forward | cagagccaacgtcaagca | 129 bp |
| (NM_013655) | reverse | aggtactcttggatccac |  |
| SDF1(human) | forward | ggaacctgaacccctgctgtg | 154 bp |
| (NM_003467) | reverse | actgggtttgtgattgcctctgaa |  |
| β-Actin | forward | tatgccaacacagtgctgtctgg | 207 bp |
|  | reverse | tactcctgcttgctgatccacat |  |
| 18S | forward | gcaattattccccatgaacg | 121 bp |
|  | reverse | ggcctcactaaaccatccaa |  |
