# Supplementary material for: Development of a Unique Small Molecule Modulator of CXCR4
Source: PLoS One. 2012 Apr 2;7(4):e34038. doi: 10.1371/journal.pone.0034038 (PMC3317778; doi:10.1371/journal.pone.0034038)
Supplement: Data S1 — Synthesis and characterization of MSX-122. (DOCX) [file pone.0034038.s002.docx]

**Data S1**

***Synthesis of MSX-122***: To a mixture of terephthaldicarboxaldehyde (5.36 g, 40 mmol), 2-amino-pyrimidine (7.82 g, 82 mmol), acetic acid (4.7 mL, 80 mmol) and 4Å molecular sieve (20 g) in 1,2-dichloroethane (250 mL) was dropwise added sodium triacetoxyborohydride (25.43 g, 120 mmol) at room temperature. After being stirred for 24 hours under an argon atmosphere, the solvent was removed under reduced pressure. The resulting residue was further washed by hot water and hot methanol consecutively to give a pale white solid which was dissolved in acetic acid (50 mL). The insoluble molecular sieve was filtered off and the acetic acid solution was concentrated to give a white solid, which was neutralized by 10% aqueous NaOH and extracted by ethyl acetate (3 × 50 mL). The combined organic phases were dried over MgSO_4_, filtered and concentrated under reduced pressure to give pure MSX-122 as a white solid (2.92 g, 25% yield)

***Characterization of MSX-122***: mp: 211-213 ^o^C; TLC (Ethyl acetate): R_f_ = 0.32; ^1^H NMR (400 MHz, DMSO-*d*_6_): δ 8.24 (d, *J* = 4.8 Hz, 4H), 7.65 (t, *J* = 6.4 Hz, 2H), 7.21 (s, 4H), 6.54 (t, *J* = 6.8 Hz, 2H), 4.43 (d, *J* = 6.4 Hz, 4H); ^13^C NMR (100 MHz, DMSO-*d*_6_): δ 162.26, 157.95, 138.59, 126.86, 110.15, 43.62; IR (neat): 3235, 3013, 1595, 1532, 1452, 1414, 1361, 1338, 1280, 1120, 1072, 846, 797, 714, 643 cm^-1^; HRMS (m/z): [M+1]^+^ calculated for C_16_H_17_N_6_ 293.1515, found 293.1505; analysis (calcd., found for C_16_H_16_N_6_): C (65.74, 65.43), H (5.52, 5.47); N (28.75, 28.73); Its salt form, MSX-122ms, was generated by carefully dissolving methane sulfonic acid (10.0 ml) in methanol (20 ml) at 0 ^o^C. To the solution, MSX-122 (818.6 mg, 2.8 mmol) was added carefully and the resulting mixture was stirred for 30 min at 0 ^o^C. After that, diethyl either was added dropwise to give white precipitate. After all precipitate was formed, the mixture was refrigerated (-7 ^o^C) for 2-4 hours and the white precipitate was collected and dried to give MSX-122ms (1.59g, 98% yield) as a pale white solid: mp:198-201 ^o^C; ^1^H NMR (400 MHz, D_2_O): δ 8.53 (br, 4H), 7.38 (s, 4H), 7.02 (t, *J* = 5.2 Hz, 2H), 4.72 (s, 4H), 2.77 (s, 7.5H); ^13^C NMR (100 MHz, D_2_O): δ 154.07, 136.01, 127.73, 110.24, 44.45, 38.51; analysis (calcd., found for C_16_H_16_N_6_•2.5CH_3_SO_3_H): C (41.72, 41.31), H (4.92, 4.91), N (15.78, 15.57).
