# Supplementary material for: Development of a Unique Small Molecule Modulator of CXCR4
Source: PLoS One. 2012 Apr 2;7(4):e34038. doi: 10.1371/journal.pone.0034038 (PMC3317778; doi:10.1371/journal.pone.0034038)
Supplement: Data S2 — Structure of various anti-CXCR4 compounds. (DOCX) [file pone.0034038.s003.docx]

**Data S2**
