# Supplementary material for: Development of a Unique Small Molecule Modulator of CXCR4
Source: PLoS One. 2012 Apr 2;7(4):e34038. doi: 10.1371/journal.pone.0034038 (PMC3317778; doi:10.1371/journal.pone.0034038)
Supplement: Data S3 — Summary of animal experiments for three metastasis models. (DOCX) [file pone.0034038.s004.docx]

**Data S3**

| Metastatic models | Group | #of mice per group | Cell lines | route of cell injection | MSX-122 dose | vehicle |
| --- | --- | --- | --- | --- | --- | --- |
| Breast cancer | Two groups: control vs. MSX-122-treated | 10 | MDA-MB-231 | 1.5x10^6^ cells tail vein injection | 4 mg/kg, i.p. daily, MSX-122ms | PBS |
| SCCHN cancer |  | 10 | 686LN-Ms |  | 10 mg/kg, i.p. daily, MSX-122ms | PBS |
| Uveal melanoma |  | 10 | OMM2.3 | Inoculation to right eye (orthotopic)  1x10^6^ | 10 mg/kg, i.p. daily, MSX-122 | 45% cyclodextran |
